# Attenuation of cGAS/STING activity during mitosis

**DOI:** 10.26508/lsa.201900636

**Published:** 2020-07-13

**Authors:** Brittany L Uhlorn, Eduardo R Gamez, Shuaizhi Li, Samuel K Campos

**Affiliations:** 1 Cancer Biology Graduate Interdisciplinary Program, The University of Arizona, Tucson, AZ, USA; 2 Department of Physiology, The University of Arizona, Tucson, AZ, USA; 3 Department of Immunobiology, The University of Arizona, Tucson, AZ, USA; 4 BIO5 Institute, The University of Arizona, Tucson, AZ, USA; 5 Department of Molecular and Cellular Biology, The University of Arizona, Tucson, AZ, USA

## Abstract

The innate immune system recognizes cytosolic DNA associated with microbial infections and cellular stress via the cGAS/STING pathway, leading to activation of phospho-IRF3 and downstream IFN-I and senescence responses. To prevent hyperactivation, cGAS/STING is presumed to be nonresponsive to chromosomal self-DNA during open mitosis, although specific regulatory mechanisms are lacking. Given a role for the Golgi in STING activation, we investigated the state of the cGAS/STING pathway in interphase cells with artificially vesiculated Golgi and in cells arrested in mitosis. We find that whereas cGAS activity is impaired through interaction with mitotic chromosomes, Golgi integrity has little effect on the enzyme’s production of cGAMP. In contrast, STING activation in response to either foreign DNA (cGAS-dependent) or exogenous cGAMP is impaired by a vesiculated Golgi. Overall, our data suggest a secondary means for cells to limit potentially harmful cGAS/STING responses during open mitosis via natural Golgi vesiculation.

## Abstract (precis)

To limit deleterious interferon responses with each cell division and open mitosis, cGAS/STING activities are shown to be dampened-with Golgi vesiculation preventing activation of STING.

## Introduction

Cells possess intrinsic sensory pathways as part of the innate immune system to detect microbial infection or other physiological insults (1). Foreign nucleic acids are often recognized as pathogen-associated molecular patterns through a number of pattern recognition receptors (2), causing activation of NFκB-dependent inflammatory cytokine responses and/or IRF3/7-dependent type-I interferon (IFN-I) responses (1, 3). The cGAS/STING pathway is recognized as a central component of innate immunity for cytosolic DNA recognition and downstream IFN-I responses (4, 5, 6, 7, 8). Cytosolic DNA is recognized by the enzyme cGAS, triggering production of the cyclic dinucleotide 2′,3′-cGAMP (9). STING, a transmembrane ER protein (10, 11), is activated by direct binding to cGAMP (12).

Upon activation by cGAMP at the ER, dimeric STING undergoes a conformational change (13) and traffics to the Golgi, a prerequisite for assembly of the STING/TBK1/IRF3 complex and downstream IFN-I responses (14). cGAMP-dependent STING recruitment of TBK1 (15) can lead to phosphorylation of IRF3 and NFκB, stimulating both IFN-I and proinflammatory cytokine responses (10, 16, 17). Trafficking of STING to the Golgi is regulated by several host factors, including iRHOM2-recruited TRAPβ (18), TMED2 (19), STIM1 (20), TMEM203 (21), and ATG9A (22). STING activation at the Golgi requires palmitoylation (23) and ubiquitylation (24, 25), allowing for assembly of oligomeric STING and recruitment of TBK1 and IRF3 (26, 27, 28). STING also interacts with the ER adaptor SCAP at the Golgi to facilitate recruitment of IRF3 (29). In addition to innate defense against microbial infections, cGAS/STING is involved in cellular responses to DNA damage and replicative/mitotic stress (5, 30, 31, 32, 33, 34, 35, 36). DNA damage, replicative stress, chromosomal instability, and mitotic errors can lead to the formation of micronuclei which can trigger antiproliferative IFN-I and senescence responses via cGAS/STING (37).

Unabated activation of cGAS/STING can lead to harmful autoinflammatory and senescence responses, exemplified by type-I interferonopathies associated with mutations in STING (38, 39, 40, 41) or mutations in the DNases TREX1 and DNASE2 that normally clear cells of cGAS-stimulatory DNA (42, 43, 44, 45). Given the harmful effects of cGAS/STING hyperactivation, cells need regulatory mechanisms to avoid self-stimulation of cGAS/STING during mitosis. Cytosolic compartmentalization of cGAS was initially proposed as a mechanism, but nuclear chromosomes and cytosolic compartments mix upon mitotic nuclear envelope breakdown (NEBD), suggesting a more elaborate means of cGAS/STING attenuation during cell division.

The chromatinized nature of cellular genomic DNA has been proposed to mitigate cGAS/STING activity, with histones structurally marking DNA as “self.” This is an attractive model as many DNA viruses sensed by cGAS/STING upon initial entry, trafficking, and uncoating (before viral DNA replication) contain either naked, unchromatinized dsDNA (e.g., herpesviridae (46, 47)) or DNA that is packaged with non-histone viral core proteins (e.g., adenoviridae, poxviridae, and asfarviridae (48, 49, 50, 51, 52)).

Because cGAS localizes to condensed chromosomes upon NEBD (35, 53), others have asked whether cGAS is activated by chromosomes, and if not, what mechanisms exist to prevent such self-activation. Recent studies have revealed that i) chromosome-bound cGAS is tightly tethered to chromatin, potentially via interactions with H2a/H2b dimers, ii) chromatin interaction does not involve the DNA-binding domains of cGAS required for “typical” activation by dsDNA, and iii) that chromosome binding results only in weak activation of cGAS with relatively low production of cGAMP (34, 53, 54).

Given the importance of the Golgi in STING-dependent activation of IRF3, we hypothesize a parallel mechanism for cGAS/STING regulation during open mitosis–Golgi vesiculation (55, 56). Here, we find that chemical dispersal of the Golgi abrogates cGAS/STING-dependent phospho-IRF3 responses to transfected DNA. Furthermore, we show that cGAS/STING activity in response to transfected DNA is diminished during open mitosis, correlating with the vesiculated state of the mitotic Golgi. This Golgi-dependent weakening of cGAS/STING responses to transfected DNA occurs at the level of STING, as cGAS activity is down-regulated upon mitotic chromosome binding but largely unaffected by Golgi integrity. The vesiculated state of the mitotic Golgi may, therefore, provide an additional safeguard mechanism, ensuring that potentially harmful cGAS/STING responses to self-DNA are minimized during cell division.

## Results and Discussion

### Human keratinocytes respond to foreign DNA via cGAS/STING

We chose to investigate activity of the cGAS/STING pathway in HaCaTs, a spontaneously immortalized human keratinocyte cell line (57). This cell line is a good model for primary keratinocytes that serve a barrier function and as host cells for a number of viral infections, including papillomaviruses, herpesviruses, mosquito-transmitted alphaviruses and flaviviruses (58, 59, 60, 61, 62, 63, 64), and numerous bacterial and fungal pathogens (65, 66). Although prior work has shown that HaCaT cells express an intact cGAS/STING pathway (60, 67, 68, 69, 70, 71), we sought to ensure the cGAS/STING pathway was functional and responsive in our HaCaT line and that this pathway was the primary mode of IRF3 phosphorylation in response to foreign DNA.

**Figure 1.**
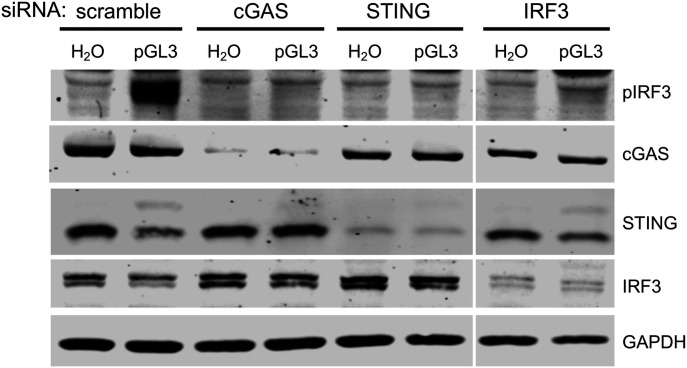
Human keratinocytes respond to foreign DNA via cGAS/STING. HaCaTs were transfected with siRNAs for 16 h, followed by transfection with 500 ng pGL3 or water for 90 min. Lysates were analyzed for cGAS/STING component knockdown and pathway activity by SDS–PAGE and Western blot. Source data are available for this figure.

HaCaTs were transfected with siRNAs targeting the cGAS/STING pathway and subsequently transfected with endotoxin-free dsDNA plasmid pGL3 (Fig 1). IRF3 was phosphorylated (pIRF3) in response to DNA transfection, exemplifying the ability of exogenous dsDNA to activate the cGAS/STING pathway in HaCaTs. IRF3 phosphorylation was impaired when pGL3 was transfected after siRNA knockdown of cGAS, STING, or IRF3, confirming that HaCaT cells use the cGAS/STING pathway as the primary mechanism of activating a pIRF3 response to foreign DNA.

### Golgi vesiculation but not fragmentation impairs cGAS/STING activity at the level of STING

**Figure 2.**
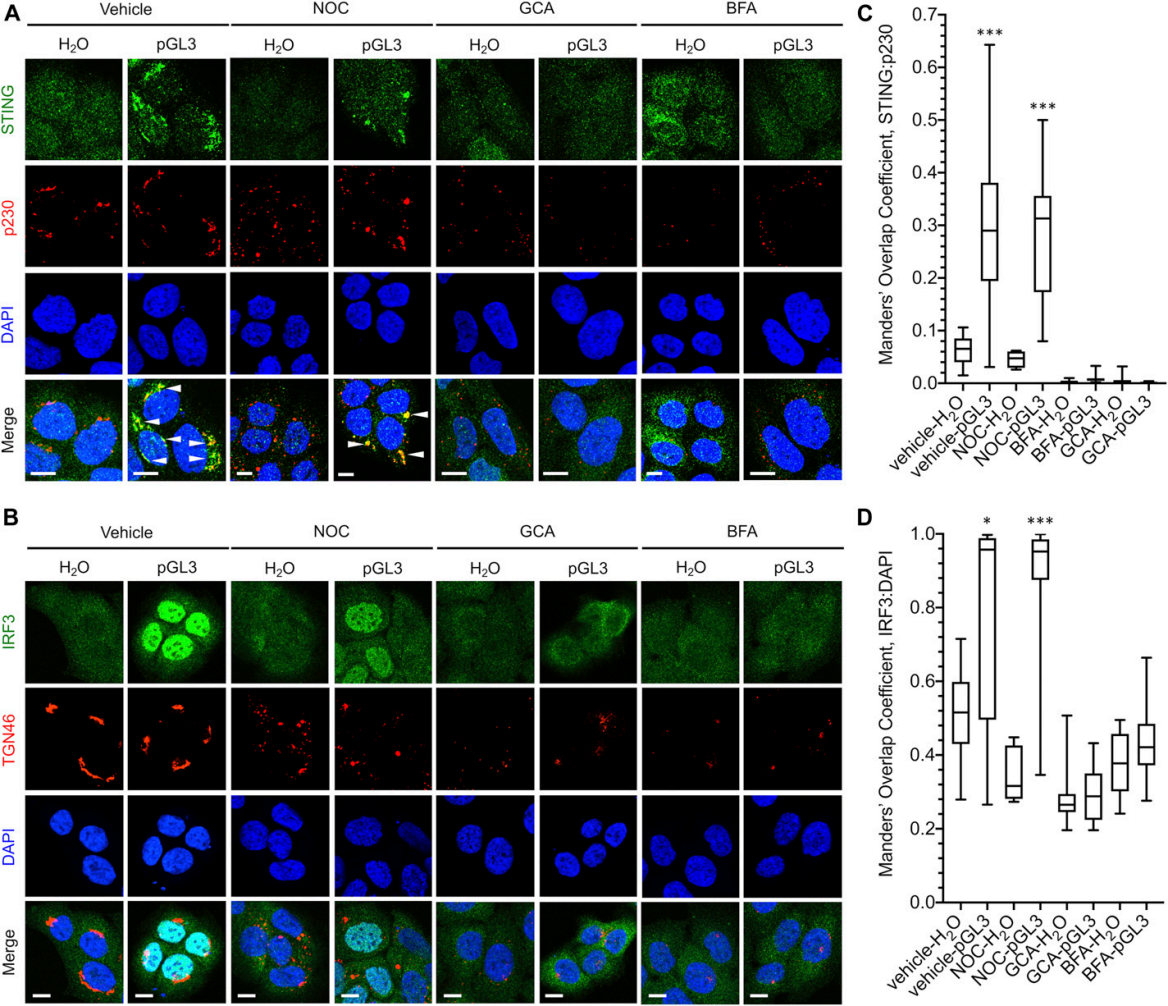
Effects of Golgi disruption on DNA-dependent subcellular localization of STING and IRF3. HaCaT cells were pretreated with vehicle, NOC, GCA, or BFA before a 90-min transfection with H_2_O or pGL3. **(A, B)** Cells were fixed and stained for (A) STING and p230 or (B) IRF3 and TGN46 before DAPI staining. **(A, B)** Representative micrographs are shown in (A, B), white arrowheads indicate overlap. **(C, D)** Manders’ overlap coefficients from multiple micrographs were plotted for (C) STING:p230 and (D) IRF3:DAPI. *P < 0.01, ***P < 0.0001. Scale bars = 10 μm.

**Figure 3.**
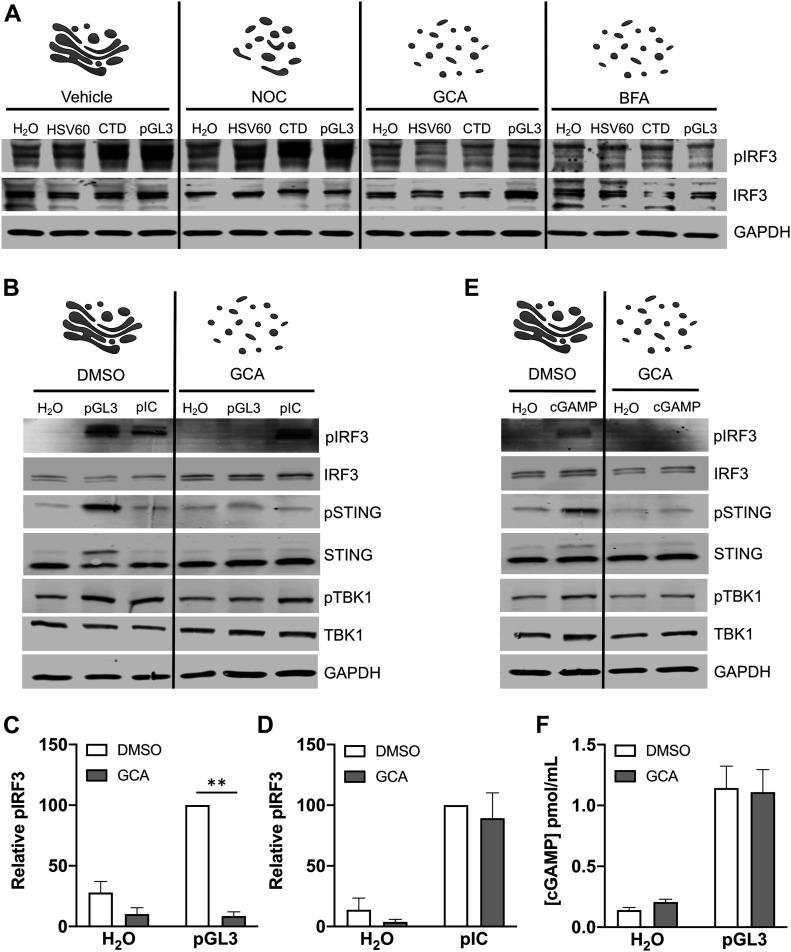
Effects of Golgi disruption on cGAS/STING activity. **(A)** Cells were treated with vehicle, nocodazole (NOC), golgicide A (GCA), or brefeldin A (BFA) for 1 h prior and during a 90-min transfection with HSV-60 oligonucleotide, calf-thymus DNA (CTD), or plasmid pGL3. **(B, C, D)** Transfection of vehicle- or GCA-treated cells with pGL3 or pIC, and (C, D) densitometric quantification of pIRF3 blots. **P < 0.001, n = 5 biological replicates. **(E)** Vehicle- or GCA-treated cells were treated with H_2_O or 12.5 μg cGAMP for 90 min before SDS–PAGE and Western blot for cGAS/STING pathway components. **(F)** cGAMP production in vehicle- and GCA-treated cells upon pGL3 transfection. n = 3 biological replicates, with n = 2 technical replicates each. Source data are available for this figure.

Activated STING traffics to the Golgi to oligomerize and complex with TBK1/IRF3. Because Golgi trafficking is critical for STING/TBK1/IRF3 complex assembly and activation, we hypothesized that a vesiculated Golgi would prevent cGAS/STING from responding to foreign DNA. To test this idea, we used the Golgi-disrupting compounds nocodazole (NOC), golgicide A (GCA), and brefeldin A (BFA). At high dose, NOC treatment causes reversible Golgi fragmentation and redistribution to ER-exit sites (ERES) (72), whereas GCA and BFA treatment cause more drastic Golgi vesiculation and dispersal by targeting the Arf1 guanine nucleotide exchange factor GBF1 (73, 74). Indeed, NOC treatment of HaCaT cells caused a pronounced fragmentation of the Golgi as seen by immunofluorescence (IF) microscopy for trans-Golgi markers p230 and TGN46, whereas GCA and BFA treatment completely vesiculated the Golgi apparatus (Fig 2A and B), mimicking the dispersed mitotic Golgi. As expected, transfection of pGL3 resulted in clustering of STING with p230- and TGN46-positive Golgi membranes and nuclear import of IRF3, indicative of pathway activation (Fig 2A–D). Addition of GCA or BFA completely abrogated activation of STING and IRF3, whereas NOC had minimal effect, suggesting that whereas a fragmented Golgi can support pGL3-stimulated STING/IRF3 activation, a dispersed Golgi cannot (Fig 2A–D). Likewise, IRF3 phosphorylation was induced upon transfection of HSV-60 oligonucleotide DNA, calf-thymus DNA (CTD), or pGL3 plasmid in HaCaTs with intact or fragmented Golgi; however, GCA- or BFA-mediated Golgi vesiculation impaired DNA-dependent IRF3 phosphorylation (Fig 3A), in agreement with prior literature (75).

To determine if the requirement for intact Golgi was specific for cGAS/STING or involved a more broad inhibition of IRF3 phosphorylation, we investigated activation of the RIG-I–like receptors (RLRs). The dsRNA-mimic polyinosinic-polycytidylic acid (pIC) stimulates RLR-family members which recognize intracellular viral RNA products through the mitochondria-resident adaptor protein MAVS to elicit NFκB inflammatory and IRF3/7-dependent IFN-I responses (76, 77). pGL3-dependent pSTING, pTBK1, and pIRF3 responses were abolished by Golgi dispersal (Fig 3B and C). In contrast, transfection of pIC elicited pTBK1 and pIRF3 responses regardless of GCA treatment (Fig 3B and D), suggesting that the cGAS/STING pathway, but not the RLR pathway, is regulated by Golgi morphology. Exogenous stimulation of HaCaT cells with the STING ligand cGAMP was sensitive to GCA-mediated Golgi disruption (Fig 3E), and GCA had no effect on cGAMP production in response to pGL3 transfection (Fig 3F), indicating that Golgi dispersal blocked the pathway downstream of cGAS.

**Figure 4.**
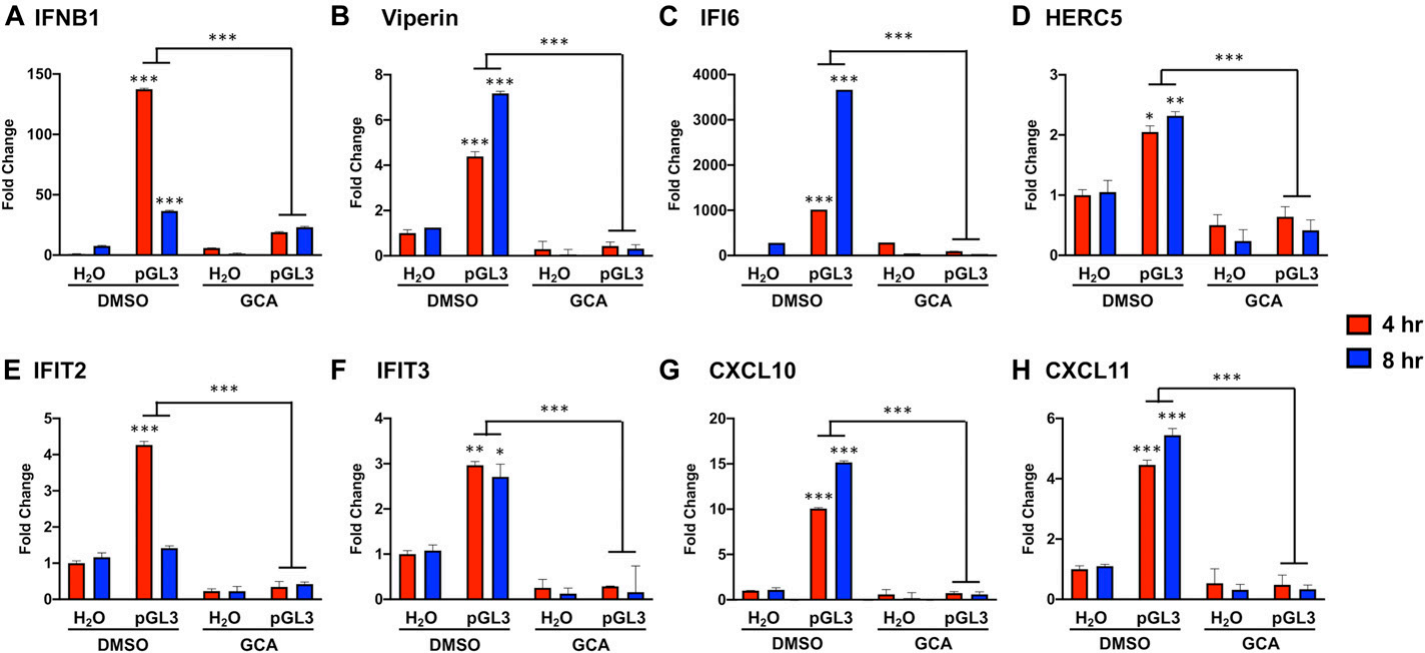
Golgi vesiculation impairs DNA-dependent IFN, ISG, and chemokine gene transcription. HaCaT cells were treated with GCA or vehicle for 1 h before a 90-min pGL3 transfection. **(A, B, C, D, E, F, G, H)** Transcript levels of (A) IFNB1, (B, C, D, E, F) ISGs, and (G, H) chemokine genes were measured via RT-qPCR and normalized to TATA-binding protein. *P < 0.01, **P < 0.001, ***P < 0.0001, for comparisons of 4 and 8 h DMSO-treated pGL3 groups to 4 and 8 h DMSO-treated H_2_O controls and for 4 and 8 h DMSO-treated pGL3 groups to 4 and 8 h GCA-treated pGL3 groups, n = 3 technical replicates.

The GCA-induced repression of cGAS/STING activation was also reflected when measuring pGL3-dependent downstream transcriptional responses. RT-qPCR at 4 and 8 h post-pGL3 transfection revealed that induction of IFNB1 (Fig 4A), the ISGs Viperin, IFI6, HERC5, IFIT2, and IFIT3 (Fig 4B–F), and chemokines CXCL10 and CXCL11 (Fig 4G and H) was significantly dampened in the presence of GCA. Overall, these data show that cGAS/STING activity is blunted at the level of STING upon Golgi vesiculation, similar to what may occur during mitosis.

### cGAS/STING activity is attenuated during mitosis

We next investigated the impact of natural mitotic Golgi vesiculation on the ability of cGAS/STING to sense and respond to exogenous DNA. Secretory ER to Golgi traffic is blocked during mitosis (78, 79, 80, 81). Golgi integrity is dependent on cargo transport from ERES, and mitotic arrest of COPII-dependent ERES traffic causes Golgi dispersal (82, 83). As assembly of the activated STING/TBK1/IRF3 complex requires STING transport from ERES to the Golgi (14), we hypothesized that mitotic Golgi dispersal and inactivation of ERES would blunt cGAS/STING responses.

**Figure 5.**
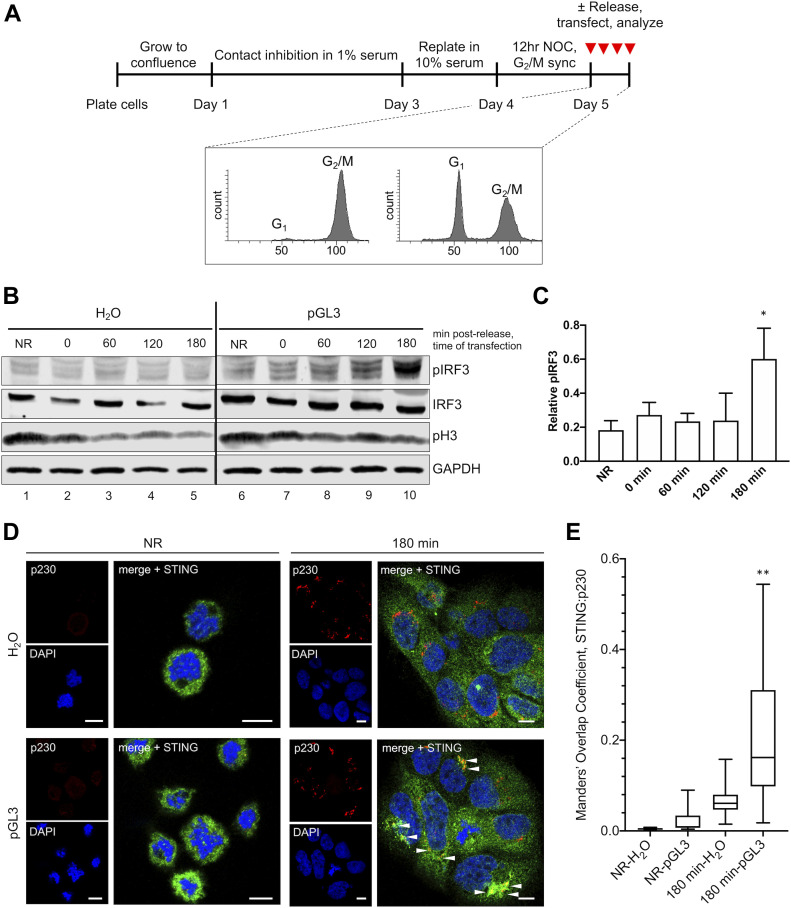
cGAS/STING activity is attenuated during mitosis. **(A)** Cell synchronization method. Cells were pre-synchronized in G_1_ by contact inhibition and growth in low serum. Cells were then released by replating at subconfluence in 10% serum and synchronized in prometaphase with NOC. Cell cycle analysis by propidium iodide staining. Cells were transfected with pGL3 for 90 min at various times post-release from NOC. **(B)** pIRF3 activation in response to pGL3 was dampened when arrested cells were transfected, gradually returning as cells completed mitosis and returned to interphase. **(C)** Densitometric quantification of relative pIRF3 increase in response to pGL3 transfection across multiple blots. *P < 0.05, for comparison of 180 min to NR groups, n = 4 independent biological replicates. **(D)** Golgi localization of STING was impaired when arrested cells (NR) were transfected, but was restored upon return to interphase (180 min). **(E)** Manders’ overlap coefficients from multiple micrographs were plotted for STING:p230, **P < 0.001, scale bars = 10 μm.

We devised a method to synchronize cells at prometaphase (Fig 5A). Briefly, cells were cultured at 100% confluence for 48 h in 1% serum, leading to quiescent arrest in G_0_. Cells were released by replating in 10% serum, allowing for G_1_ reentry and progression to S phase. At 24 h post-G_0_ release, cells were synchronized at prometaphase with low-dose NOC for 12 h. Upon NOC washout, synchronized cells progressed through mitosis, returning to G_1_ within 3 h. Propidium iodide (PI) staining showed cells enriched at G_2_/M after NOC treatment, and the most cells back in G_1_ by 3 h post-NOC release (Fig 5A). Phosphorylated histone H3 (pH3) was enriched in NOC-synchronized cells, decreasing as cells returned to G_1_ (Fig 5B).

We used this scheme to assess mitotic cGAS/STING responses to pGL3 transfection. Cells were transfected while in the prometaphase after the 12 h NOC sync (non-released group, NR), or at 0, 60, 120, or 180 min post-release, and cGAS/STING activity was assessed 90 min post-transfection. Cells arrested at the prometaphase mounted a very weak pIRF3 response to pGL3 transfection compared with cells which had returned to G_1_ after a 3 h release (Fig 5B, compare lanes 6–10). These arrested cells had condensed chromosomes with dispersed Golgi (Fig 5D, NR). STING had a cytosolic but granular distribution in arrested cells, which did not change upon pGL3 transfection. In contrast, STING clearly localized to p230-positive Golgi structures upon pGL3 transfection of G_1_ cells (Fig 5D and E). On average, across four independent synchronization experiments, pGL3-dependent phosphorylation of IRF3 was attenuated during mitosis, only becoming robust at 180 min (Fig 5C), when the bulk population of cells reached G_1_ and had intact Golgi with clear STING localization upon pGL3 transfection (Fig 5D and E).

### cGAS and STING are nonresponsive during mitosis

Recent work has shown that upon NEBD, cGAS localizes to mitotic chromosomes via direct binding to H2a/H2b dimers (53). However, this binding is not via the DNA-binding domains of cGAS that underlie DNA-dependent activation, resulting in a relatively low production of cGAMP. Thus, chromatin appears to blunt cGAS activation (34, 53), suggesting a means for the cell to avoid cGAS-driven IFN responses to self-chromosomes during open mitosis. One unexplored aspect is whether chromatin-bound cGAS, or the pool of cGAS that might remain cytosolic during open mitosis, would still be responsive to foreign (naked) DNA—and whether that activation would then cause IRF3 phosphorylation.

**Figure 6.**
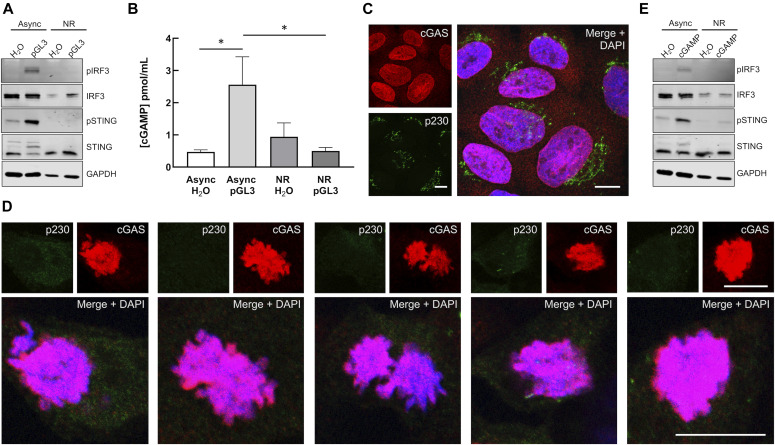
cGAS and STING are nonresponsive during mitosis. **(A)** Mitotic phospho-IRF3 and phospho-STING responses to pGL3. Asynchronous and arrested cells were stimulated by transfection of 500 ng pGL3 or water for 90 min before Western blots. **(B)** Mitotic cGAMP responses to pGL3. Asynchronous and arrested cells were transfected with 500 ng pGL3. Non-internalized transfection complexes were removed by media change 2 h after the initial transfection. 5 h post-transfection, cGAMP levels were measured by ELISA. *P < 0.05, n = 2 biological replicates, with n = 2 technical replicates each. **(C, D)** cGAS subcellular localization and Golgi morphology in asynchronous (C) and arrested (D) HaCaT cells. Scale bars = 10 μm. **(E)** Mitotic phospho-IRF3 and phospho-STING responses to exogenous cGAMP. Asynchronous and arrested cells were treated with 25 μg/ml 2′-3′cGAMP for 2 h before Western blots. Source data are available for this figure.

We observed only low activation of IRF3 in response to pGL3 transfection in NOC-arrested prometaphase cells (Figs 5B and C and 6A). To determine if the dampening of the pathway in these arrested cells was at the level of cGAS or STING, we measured cGAMP production in response to pGL3 transfection and examined cGAS subcellular distribution. Similar to pIRF3, cGAMP production was blunted in transfected arrested cells (NR) compared with asynchronous interphase cells (Fig 6B). Confocal microscopy of asynchronous cells revealed that cGAS was mostly nuclear, although some signal was evident within the cytosol (Fig 6C), in agreement with a recent report (54). Within arrested cells, the vast majority of cGAS was chromatin bound and Golgi were well dispersed (Fig 6D). These results agree with recent work showing that cGAS is predominantly nuclear, regardless of cell cycle phase or activation status (54). Combined, our data suggest that chromatin-bound cGAS is unable to produce a robust cGAMP response to either chromosomes or transfected DNA.

Although we detected slightly elevated levels of cGAMP in unstimulated arrested versus asynchronous cells (Fig 6B), the subtle difference was not significant, consistent with recent work showing cGAS generates only low levels of cGAMP upon activation by chromatin (34, 53). Mitotic cells could potentially take up exogenous cGAMP via SLC19A1 (84, 85) or LRRC8 (86) transporters, or directly from neighboring cells (87, 88). Recent work has revealed a primordial role for cGAMP-dependent STING activation in triggering autophagy through WIPI2/ATG5 in a TBK1/IRF3-independent manner (75). These Golgi-derived autophagosomes promote clearance of cytosolic DNA and incoming DNA viruses such as HSV1 (75). Although mitotic cells appear to avoid classical induction of autophagy via CDK1 phosphorylation of ATG13, ULK1, and ATG14 (89, 90), downstream STING-dependent activation of autophagophore formation/elongation via WIPI2/ATG5 during open mitosis could be deleterious to daughter cell survival (91, 92).

Considering the potential for activation of STING by low levels of endogenous cGAMP or uptake of exogenous cGAMP, we assessed whether mitotic Golgi vesiculation would prevent STING activation by exogenous cGAMP. Neither transfection of pGL3 nor addition of exogenous cGAMP stimulated pIRF3 or pSTING in arrested cells (Fig 6E), suggesting that Golgi vesiculation reinforces cGAS inactivation as a secondary barrier to cGAS/STING activation during open mitosis. During revision of this manuscript, an article was published describing how cGAS is phosphorylated and inactivated by the kinase Cdk1-cyclin B during mitosis (93). Using different cell types, their findings largely agree with what we observe herein—that chromosome-bound cGAS is inactive to stimulation by exogenous transfected DNA in mitosis and that STING is nonresponsive to exogenous cGAMP in mitotic cells. Interestingly, when mitotic cGAS inactivation was blunted by the use of the Cdk1 inhibitor RO-3306, despite increased cGAMP levels or addition of exogenous cGAMP, there was still lack of activated pSTING and pIRF3 (93), consistent with our data suggesting pathway inhibition by a vesiculated Golgi. Fragmentation and vesiculation of the Golgi is a natural G_2_/M checkpoint for mitotic progression (55, 94, 95, 96), thus we are unable to experimentally prevent Golgi dispersal during mitosis to determine if cGAMP-dependent STING activity would then be restored.

Given that cGAS/STING-dependent elevation of pIRF3 during mitosis (particularly during prolonged mitosis) can induce apoptosis (53) and activated STING can induce a potentially harmful autophagic response (75), a parallel dampening mechanism like Golgi dispersal likely serves an important role to limit potentially harmful cGAS/STING signaling during open mitosis. Furthermore, many other viruses and bacteria induce dramatic alteration of Golgi membranes during infection (97, 98, 99, 100, 101). Microbial alteration of Golgi integrity may be an important unrecognized tactic to blunt host cGAS/STING responses.

## Materials and Methods

### Tissue culture

HaCaT cells were grown in high-glucose DMEM (11965-092; Gibco) supplemented with 10% FBS (A31606-02; Gibco) and antibiotic-antimycotic (15240062; Thermo Fisher Scientific). Cells were cultured at 37°C with 5% CO_2_ and passaged every 2–3 d to maintain subconfluence.

### Nucleic acid transfections

HaCaTs were plated at 60,000 cells per well in a 24-well plate. Cells were transfected with 500 ng dsDNA oligonucleotide HSV-60 (tlrl-hsv-60n, 60 bp; InvivoGen), calf-thymus DNA (D4764, >20 kb; Sigma-Aldrich), or endotoxin-free pGL3 (E1751, 5.3 kb; Promega), or 500 ng high molecular weight poly(I:C) (tlrl-pic, 1.5–8 kb; InvivoGen) using Lipofectamine 2000 (11668; Thermo Fisher Scientific) in OptiMEM (Life Technologies). At various time points post-transfection, cells were washed once with PBS and lysed in 1× RIPA lysis buffer (50 mM Tris–HCl, pH 8.0, 150 mM NaCl, 1% NP40, 0.5% sodium deoxycholate, and 0.1% SDS), supplemented with 1× reducing SDS–PAGE loading buffer (62.5 mM Tris, pH 6.8, 10% glycerol, 2% SDS, 0.5% bromophenol blue, and 5% β-mercaptoethanol), 1× protease inhibitor cocktail (P1860; Sigma-Aldrich), 1 mM PMSF, and 1× PhosSTOP phosphatase inhibitor cocktail (04906845001; Roche). Samples were boiled for 5 min at 95°C and stored at −80°C until separated and analyzed by SDS–PAGE.

### siRNA experiments

Pooled scramble (sc-37007), cGAS (sc-95512), STING (sc-92042), and IRF3 (sc-35710) siRNA duplexes were obtained from Santa Cruz Biotechnologies. HaCaTs were plated at 30,000 cells per well in a 24-well plate with Ab/Am-free DMEM/10% FBS. The following day, cells were washed twice with PBS and the PBS replaced with OptiMEM. Cells were transfected with 50 nM siRNA using Lipofectamine RNAiMax (13778150; Life Technologies). At 16 h post-siRNA transfection, cells were washed twice with PBS and the PBS was replaced with Ab/Am-free DMEM/10% FBS. Cells were transfected with pGL3 24 h post-siRNA transfection, as described above. At 90 min post-transfection, samples were collected for Western blotting as described above.

### Golgi disruption

HaCaTs were plated at 60,000 cells per well on glass coverslips in 24-well plates. The following day, cells were treated with 5 μM nocodazole (sc-3518; Santa Cruz), 10 μM golgicide A (sc-215103; Santa Cruz), or 150 nM brefeldin A (B6542; Sigma-Aldrich), or DMSO vehicle for 1 h before further experimental treatments, and drugs were left on for the duration of these experiments.

### Cell synchronization

HaCaTs were plated at 7.5 million cells on a 10-cm dish in 10% FBS/DMEM and allowed to reach 100% confluence. The day after plating, the medium was changed to 1% FBS/DMEM and cells remained under contact inhibition at low-serum conditions for 48 h to synchronize at G_0_. Cells were then replated at 30,000 cells per well on 24-well plates with or without glass coverslips in 10% FBS/DMEM and allowed to progress through the S phase. After 24 h, the cells were treated with 50 ng/ml nocodazole (sc-3518; Santa Cruz) for 12 h to synchronize at the prometaphase, at which point they were released with two PBS washes and incubated in 10% FBS/DMEM. Cells were transfected with pGL3 as described above, and either harvested for Western blotting or prepared for IF or cell cycle analysis as described.

### Cell cycle analysis

Cell cycle status was analyzed by PI incorporation and flow cytometry. HaCaTs synchronized in the manner described above. At various time points during the synchronization and release from the prometaphase, cells were collected by trypsinization and pelleted at 500*g* for 10 min at 4°C. The pellet was resuspended in ice-cold 70% ethanol to fix the cells and stored at −20°C until ready for PI staining. Fixed cells were pelleted at 1,000*g* for 15 min at 4°C, resuspended in PBS, pH 7.4, containing 40 μg/ml PI and 500 μg/ml RNase A, and incubated at 37°C for 30 min. PI-stained cells were analyzed using the BD Biosciences FACSCanto-II flow cytometer and Diva 8.0 software.

### Immunofluorescent staining

HaCaTs were plated at 60,000 cells per well on glass coverslips in 24-well plates. The following day, cells were treated and transfected as described above. For mitotic sync experiments: HaCaTs were synchronized to prometaphase as described above. For all experiments, after transfection, cells were fixed with 2% paraformaldehyde/PBS for 10 min at RT and permeabilized with 0.2% Triton X-100/PBS for 10 min at RT. Samples were blocked in 4% BSA/1% goat serum/PBS overnight at 4°C. Rabbit polyclonal anti-TGN46 (T7576. 1:500; Sigma-Aldrich), mouse monoclonal anti-p230 (611280, 1:500; BD Biosciences), mouse monoclonal anti-IRF3 (ab50772, 1:100; Abcam), rabbit anti-cGAS (15102, 1:100; Cell Signaling), and rabbit monoclonal anti-STING (ab181125, 1:500; Abcam) were used as primary antibodies. Alexa Fluor–488, Alexa Fluor–555, and Alexa Fluor–647 labeled goat antimouse and goat antirabbit secondary antibodies (A11029, A21424, A21429, and A21236; Life Technologies) were used at 1:1,000. Samples were then stained with 4′,6-diamidino-2-phenylindole (DAPI) (D9542-10MG; Sigma-Aldrich) at 1 μg/ml for 30 s. Coverslips were mounted on glass slides with Prolong Antifade Diamond (P36970; Life Technologies) and analyzed by confocal microscopy.

### Confocal microscopy

After the preparation of IF slides, confocal microscopy was performed using a Zeiss LSM880 system with 405, 488, and 543 nm lasers. Samples were examined using an oiled 63× objective, and Z-stacks with a 0.32 μm depth per plane were taken of each image. Representative single-plane images and Z-stacks were processed with the Zen Blue software.

### Colocalization analysis

Manders’ overlap coefficients (102) for a STING:p230 and IRF3:DAPI channels within individual Z-stacks were determined using the JACoP plugin (103) on ImageJ (104). Manual thresholds were set below saturation. Individual Manders overlap coefficient values and mean values from multiple Z-stacks (each containing multiple cells), across multiple fields of view, were plotted with GraphPad Prism software.

### cGAMP stimulation

HaCaTs were plated at 60,000 asynchronous cells or 130,000 synchronous cells per well in 24-well plates. Cells were treated with 12.5 or 25 μg/ml 2′-3′cGAMP (tlrl-nacga23; Invivogen) for 2 h as indicated, then harvested for Western blotting as described above.

### SDS–PAGE and Western blotting

Samples were resolved by SDS–PAGE and transferred onto a 0.45-μm nitrocellulose membrane. Rabbit monoclonal anti-GAPDH (2118, 1:5,000; Cell Signaling), mouse monoclonal anti-IRF3 (50772, 1:100; Abcam), rabbit monoclonal anti-TBK1 (3504, 1:1,000; Cell Signaling), and rabbit monoclonal anti-STING (13647, 1:1,000; Cell Signaling) blots were blocked in 5% nonfat powdered milk dissolved in Tris-buffered saline containing 0.1% Tween (TBST). Rabbit monoclonal anti–phospho-IRF3 (Ser396 4947, 1:1,000; Cell Signaling), rabbit monoclonal anti–phospho-STING (Ser366 19781, 1:1,000; Cell Signaling), rabbit monoclonal anti–phospho-TBK1 (Ser172 5483, 1:1,000; Cell Signaling), and rabbit monoclonal anti-phospho-H3 (Ser10 3377, 1:10,000; Cell Signaling) blots were blocked in 100% Odyssey Blocking Buffer (927–40000; LI-COR). Goat antirabbit DyLight 680 (3568; Pierce), goat antimouse DyLight 680 (35518; Pierce), goat antirabbit DyLight 800 (535571; Pierce), and goat antimouse DyLight 800 (35521; Pierce) were used as secondary antibodies at 1:10,000 in either 50% Odyssey Blocking Buffer/TBST or 5% milk/TBST. Blots were imaged with the Licor Odyssey Infrared Imaging System. Band intensities were quantified by densitometry using ImageJ v1.52a (104).

### cGAMP ELISA

After Golgi vesiculation and mitotic sync experiments, cells were washed 1× with PBS and prepared for cGAMP quantification by the 2′,3′-Cyclic GAMP Direct ELISA Kit (K067-H1; Arbor Assays), following the manufacturer’s protocol. cGAMP concentrations were normalized by total protein concentration, as determined by BCA Assay (23225; Thermo Fisher Scientific).

### RT-qPCR experiments

After Golgi disruption with GCA and transfection with pGL3, total RNA was prepared from HaCaTs using the QIAGEN RNeasy Mini Kit (74104; QIAGEN). RNA was isolated at 4 and 8 h post-pGL3 transfection. In the final step, RNA was eluted in 45 μl RNase/DNase-free water. RNA samples were purified using the TURBO DNA-free Kit (AM1907; Life Technologies), and cDNA was prepared using the High Capacity cDNA Reverse Transcription Kit (4368814; Thermo Fisher Scientific). To prepare cDNA, 1 μg of RNA was used per 40 μl final reaction volume, yielding an estimated cDNA concentration of 25 ng/μl. cDNA was diluted to 3.3 ng/μl with RNase/DNase-free water before use in qPCR. Reactions with specific ISG/IFN or TATA-binding protein (TBP) housekeeper primers were prepared using the PowerUp SYBR Green Master Mix Kit (A25742; Thermo Fisher Scientific) and loaded onto a 384-well plate. The reactions were run on a QuantStudio6 Flex Real-Time PCR System (Thermo Fisher Scientific). Delta-delta-cycle threshold (ΔΔCt) was determined relative to vehicle treated samples. Viral RNA levels were normalized to TBP housekeeper and depicted as fold change over vehicle treated samples. Error bars indicate the SEM from n = 3 technical replicates. Primer sequences are as follows: TBP-for; 5′-TAAACTTGACCTAAAGACCATTGCA-3′, TBP-rev; 5′-CAGCAAACCGCTTGGGATTA-3′, IFNB1-for; 5′-CTTGGATTCCTACAAAGAAGCAGC-3′, IFNB1-rev; 5′-TCCTCCTTCTGGAACTGCTGCA-3′, viperin-for; 5′-CCAGTGCAACTACAAATGCGGC-3′, viperin-rev, 5′-CGGTCTTGAAGAAATGGCTCTCC-3′, IFI6-for; 5′-TGATGAGCTGGTCTGCGATCCT-3′, IFI6-rev; 5′-GTAGCCCATCAGGGCACCAATA-3′, HERC5-for; 5′-CAACTGGGAGAGCCTTGTGGTT-3′, HERC5-rev; 5′-CTGGACCAGTTTGCTGAAAGTGG-3′, IFIT2-for; 5′-GGAGCAGATTCTGAGGCTTTGC-3′, IFIT2-rev; 5′-GGATGAGGCTTCCAGACTCCAA-3′, IFIT3-for; 5′-CCTGGAATGCTTACGGCAAGCT-3′, IFIT3-rev; 5′-GAGCATCTGAGAGTCTGCCCAA-3′, CXCL10-for; 5′-GGTGAGAAGAGATGTCTGAATCC-3′, CXCL10-rev; 5′-GTCCATCCTTGGAAGCACTGCA-3′, CXCL11-for; 5′-AAGGACAACGATGCCTAAATCCC-3′, CXCL11-rev; 5′-CAGATGCCCTTTTCCAGGACTTC-3′.

### Statistics

Statistical analyses were performed using Prism 6 (GraphPad Software). Statistics for the pIRF3 blot densitometry from Golgi vesiculation experiments were determined by an unpaired *t* test with the Holm–Sidak correction. Statistics for the cGAMP ELISAs and pIRF3 densitometry in Fig 5 were determined by ordinary one-way ANOVA with the Tukey correction for multiple comparisons. RT-qPCR data were analyzed by two-way ANOVA with Tukey’s multiple comparison. Colocalization statistics were calculated using a two-sample unpaired *t* test as recommended for colocalization analysis (105). Error bars on graphs represent standard error of mean. Number of biological and technical replicates is noted in figure legends.

## Supplementary Material

Reviewer comments

## References

[1] Medzhitov R (2007) Recognition of microorganisms and activation of the immune response. Nature 449: 819–826. 10.1038/nature06246 17943118

[2] Takeuchi O, Akira S (2010) Pattern recognition receptors and inflammation. Cell 140: 805–820. 10.1016/j.cell.2010.01.022 20303872

[3] Stetson D, Medzhitov R (2006) Type I interferons in host defense. Immunity 25: 373–381. 10.1016/j.immuni.2006.08.007 16979569

[4] Wu J, Chen Z (2014) Innate immune sensing and signaling of cytosolic nucleic acids. Annu Rev Immunol 32: 461–488. 10.1146/annurev-immunol-032713-120156 24655297

[5] Li T, Chen Z (2018) The cGAS-cGAMP-STING pathway connects DNA damage to inflammation, senescence, and cancer. J Exp Med 215: 1287–1299. 10.1084/jem.20180139 29622565 PMC5940270

[6] Chen Q, Sun L, Chen Z (2016) Regulation and function of the cGAS-STING pathway of cytosolic DNA sensing. Nat Immunol 17: 1142–1149. 10.1038/ni.3558 27648547

[7] Tao J, Zhou X, Jiang Z (2016) cGAS-cGAMP-STING: The three musketeers of cytosolic DNA sensing and signaling. IUBMB Life 68: 858–870. 10.1002/iub.1566 27706894

[8] Stetson D, Medzhitov R (2006) Recognition of cytosolic DNA activates an IRF3-dependent innate immune response. Immunity 24: 93–103. 10.1016/j.immuni.2005.12.003 16413926

[9] Sun L, Wu J, Du F, Chen X, Chen Z (2013) Cyclic GMP-AMP synthase is a cytosolic DNA sensor that activates the type I interferon pathway. Science 339: 786–791. 10.1126/science.1232458 23258413 PMC3863629

[10] Ishikawa H, Barber G (2008) STING is an endoplasmic reticulum adaptor that facilitates innate immune signalling. Nature 455: 674–678. 10.1038/nature07317 18724357 PMC2804933

[11] Ishikawa H, Ma Z, Barber G (2009) STING regulates intracellular DNA-mediated, type I interferon-dependent innate immunity. Nature 461: 788–792. 10.1038/nature08476 19776740 PMC4664154

[12] Yin Q, Tian Y, Kabaleeswaran V, Jiang X, Tu D, Eck M, Chen Z, Wu H (2012) Cyclic di-GMP sensing via the innate immune signaling protein STING. Mol Cell 46: 735–745. 10.1016/j.molcel.2012.05.029 22705373 PMC3697849

[13] Gao P, Ascano M, Zillinger T, Wang W, Dai P, Serganov A, Gaffney B, Shuman S, Jones R, Deng L, (2013) Structure-function analysis of STING activation by c[G(2′,5′)pA(3′,5′)p] and targeting by antiviral DMXAA. Cell 154: 748–762. 10.1016/j.cell.2013.07.023 23910378 PMC4386733

[14] Ogawa E, Mukai K, Saito K, Arai H, Taguchi T (2018) The binding of TBK1 to STING requires exocytic membrane traffic from the ER. Biochem Biophys Res Commun 503: 138–145. 10.1016/j.bbrc.2018.05.199 29870684

[15] Tanaka Y, Chen Z (2012) STING specifies IRF3 phosphorylation by TBK1 in the cytosolic DNA signaling pathway. Sci Signal 5: ra20 10.1126/scisignal.2002521 22394562 PMC3549669

[16] Abe T, Barber G (2014) Cytosolic-DNA-mediated, STING-dependent proinflammatory gene induction necessitates canonical NF-kappaB activation through TBK1. J Virol 88: 5328–5341. 10.1128/jvi.00037-14 24600004 PMC4019140

[17] Honda K, Taniguchi T (2006) IRFs: Master regulators of signalling by Toll-like receptors and cytosolic pattern-recognition receptors. Nat Rev Immunol 6: 644–658. 10.1038/nri1900 16932750

[18] Luo W, Li S, Li C, Lian H, Yang Q, Zhong B, Shu H (2016) iRhom2 is essential for innate immunity to DNA viruses by mediating trafficking and stability of the adaptor STING. Nat Immunol 17: 1057–1066. 10.1038/ni.3510 27428826

[19] Sun M, Zhang J, Jiang L, Pan Y, Tan J, Yu F, Guo L, Yin L, Shen C, Shu H, (2018) TMED2 potentiates cellular IFN responses to DNA viruses by reinforcing MITA dimerization and facilitating its trafficking. Cell Rep 25: 3086–3098.e3. 10.1016/j.celrep.2018.11.048 30540941

[20] Srikanth S, Woo J, Wu B, El-Sherbiny Y, Leung J, Chupradit K, Rice L, Seo G, Calmettes G, Ramakrishna C, (2019) The Ca(2+) sensor STIM1 regulates the type I interferon response by retaining the signaling adaptor STING at the endoplasmic reticulum. Nat Immunol 20: 152–162. 10.1038/s41590-018-0287-8 30643259 PMC6340781

[21] Li Y, James S, Wyllie D, Wynne C, Czibula A, Bukhari A, Pye K, Bte Mustafah S, Fajka-Boja R, Szabo E, (2019) TMEM203 is a binding partner and regulator of STING-mediated inflammatory signaling in macrophages. Proc Natl Acad Sci U S A 116: 16479–16488. 10.1073/pnas.1901090116 31346090 PMC6697806

[22] Saitoh T, Fujita N, Hayashi T, Takahara K, Satoh T, Lee H, Matsunaga K, Kageyama S, Omori H, Noda T, (2009) Atg9a controls dsDNA-driven dynamic translocation of STING and the innate immune response. Proc Natl Acad Sci U S A 106: 20842–20846. 10.1073/pnas.0911267106 19926846 PMC2791563

[23] Mukai K, Konno H, Akiba T, Uemura T, Waguri S, Kobayashi T, Barber G, Arai H, Taguchi T (2016) Activation of STING requires palmitoylation at the Golgi. Nat Commun 7: 11932 10.1038/ncomms11932 27324217 PMC4919521

[24] Tsuchida T, Zou J, Saitoh T, Kumar H, Abe T, Matsuura Y, Kawai T, Akira S (2010) The ubiquitin ligase TRIM56 regulates innate immune responses to intracellular double-stranded DNA. Immunity 33: 765–776. 10.1016/j.immuni.2010.10.013 21074459

[25] Zhang J, Hu M, Wang Y, Shu H (2012) TRIM32 protein modulates type I interferon induction and cellular antiviral response by targeting MITA/STING protein for K63-linked ubiquitination. J Biol Chem 287: 28646–28655. 10.1074/jbc.m112.362608 22745133 PMC3436586

[26] Shang G, Zhang C, Chen Z, Bai X, Zhang X (2019) Cryo-EM structures of STING reveal its mechanism of activation by cyclic GMP-AMP. Nature 567: 389–393. 10.1038/s41586-019-0998-5 30842659 PMC6859894

[27] Ergun S, Fernandez D, Weiss T, Li L (2019) STING polymer structure reveals mechanisms for activation, hyperactivation, and inhibition. Cell 178: 290–301.e10. 10.1016/j.cell.2019.05.036 31230712

[28] Zhang C, Shang G, Gui X, Zhang X, Bai X, Chen Z (2019) Structural basis of STING binding with and phosphorylation by TBK1. Nature 567: 394–398. 10.1038/s41586-019-1000-2 30842653 PMC6862768

[29] Chen W, Li S, Yu H, Liu X, Huang L, Wang Q, Liu H, Cui Y, Tang Y, Zhang P, (2016) ER adaptor SCAP translocates and recruits IRF3 to perinuclear microsome induced by cytosolic microbial DNAs. PLoS Pathog 12: e1005462 10.1371/journal.ppat.1005462 26900919 PMC4762662

[30] Ablasser A, Chen Z (2019) cGAS in action: Expanding roles in immunity and inflammation. Science 363: eaat8657 10.1126/science.aat8657 30846571

[31] Bakhoum S, Ngo B, Laughney A, Cavallo J, Murphy C, Ly P, Shah P, Sriram R, Watkins T, Taunk N, (2018) Chromosomal instability drives metastasis through a cytosolic DNA response. Nature 553: 467–472. 10.1038/nature25432 29342134 PMC5785464

[32] Gluck S, Guey B, Gulen M, Wolter K, Kang T, Schmacke N, Bridgeman A, Rehwinkel J, Zender L, Ablasser A (2017) Innate immune sensing of cytosolic chromatin fragments through cGAS promotes senescence. Nat Cell Biol 19: 1061–1070. 10.1038/ncb3586 28759028 PMC5826565

[33] Harding S, Benci J, Irianto J, Discher D, Minn A, Greenberg R (2017) Mitotic progression following DNA damage enables pattern recognition within micronuclei. Nature 548: 466–470. 10.1038/nature23470 28759889 PMC5857357

[34] Mackenzie K, Carroll P, Martin C, Murina O, Fluteau A, Simpson D, Olova N, Sutcliffe H, Rainger J, Leitch A, (2017) cGAS surveillance of micronuclei links genome instability to innate immunity. Nature 548: 461–465. 10.1038/nature23449 28738408 PMC5870830

[35] Yang H, Wang H, Ren J, Chen Q, Chen Z (2017) cGAS is essential for cellular senescence. Proc Natl Acad Sci U S A 114: E4612–E4620. 10.1073/pnas.1705499114 28533362 PMC5468617

[36] Dou Z, Ghosh K, Vizioli M, Zhu J, Sen P, Wangensteen K, Simithy J, Lan Y, Lin Y, Zhou Z, (2017) Cytoplasmic chromatin triggers inflammation in senescence and cancer. Nature 550: 402–406. 10.1038/nature24050 28976970 PMC5850938

[37] Motwani M, Pesiridis S, Fitzgerald K (2019) DNA sensing by the cGAS-STING pathway in health and disease. Nat Rev Genet 20: 657–674. 10.1038/s41576-019-0151-1 31358977

[38] Konno H, Chinn I, Hong D, Orange J, Lupski J, Mendoza A, Pedroza L, Barber G (2018) Pro-inflammation associated with a gain-of-function mutation (R284S) in the innate immune sensor STING. Cell Rep 23: 1112–1123. 10.1016/j.celrep.2018.03.115 29694889 PMC6092751

[39] Liu Y, Jesus A, Marrero B, Yang D, Ramsey S, Sanchez G, Tenbrock K, Wittkowski H, Jones O, Kuehn H, (2014) Activated STING in a vascular and pulmonary syndrome. N Engl J Med 371: 507–518. 10.1056/nejmoa1312625 25029335 PMC4174543

[40] Picard C, Thouvenin G, Kannengiesser C, Dubus J, Jeremiah N, Rieux-Laucat F, Crestani B, Belot A, Thivolet-Bejui F, Secq V, (2016) Severe pulmonary fibrosis as the first manifestation of interferonopathy (TMEM173 mutation). Chest 150: e65–e71. 10.1016/j.chest.2016.02.682 27613991

[41] Saldanha R, Balka K, Davidson S, Wainstein B, Wong M, Macintosh R, Loo C, Weber M, Kamath V, Moghaddas F, (2018) A mutation outside the dimerization domain causing atypical STING-associated vasculopathy with onset in infancy. Front Immunol 9: 1535 10.3389/fimmu.2018.01535 30038614 PMC6047589

[42] Davidson S, Steiner A, Harapas C, Masters S (2018) An update on autoinflammatory diseases: Interferonopathies. Curr Rheumatol Rep 20: 38 10.1007/s11926-018-0748-y 29846818

[43] Stetson D, Ko J, Heidmann T, Medzhitov R (2008) Trex1 prevents cell-intrinsic initiation of autoimmunity. Cell 134: 587–598. 10.1016/j.cell.2008.06.032 18724932 PMC2626626

[44] Gray E, Treuting P, Woodward J, Stetson D (2015) Cutting edge: cGAS is required for lethal autoimmune disease in the Trex1-deficient mouse model of aicardi-goutieres syndrome. J Immunol 195: 1939–1943. 10.4049/jimmunol.1500969 26223655 PMC4546858

[45] Yoshida H, Okabe Y, Kawane K, Fukuyama H, Nagata S (2005) Lethal anemia caused by interferon-beta produced in mouse embryos carrying undigested DNA. Nat Immunol 6: 49–56. 10.1038/ni1146 15568025

[46] Sun C, Luecke S, Bodda C, Jonsson K, Cai Y, Zhang B, Jensen S, Nordentoft I, Jensen J, Jakobsen M, (2019) Cellular requirements for sensing and elimination of incoming HSV-1 DNA and capsids. J Interferon Cytokine Res 39: 191–204. 10.1089/jir.2018.0141 30855198

[47] Hare D, Collins S, Mukherjee S, Loo Y, Gale M Jr., Janssen L, Mossman K (2015) Membrane perturbation-associated Ca2+ signaling and incoming genome sensing are required for the host response to low-level enveloped virus particle entry. J Virol 90: 3018–3027. 10.1128/jvi.02642-15 26719279 PMC4810640

[48] Lam E, Stein S, Falck-Pedersen E (2014) Adenovirus detection by the cGAS/STING/TBK1 DNA sensing cascade. J Virol 88: 974–981. 10.1128/jvi.02702-13 24198409 PMC3911663

[49] Wang F, Alain T, Szretter K, Stephenson K, Pol J, Atherton M, Hoang H, Fonseca B, Zakaria C, Chen L, (2016) S6K-STING interaction regulates cytosolic DNA-mediated activation of the transcription factor IRF3. Nat Immunol 17: 514–522. 10.1038/ni.3433 27043414 PMC4917298

[50] Watkinson R, McEwan W, Tam J, Vaysburd M, James L (2015) TRIM21 promotes cGAS and RIG-I sensing of viral genomes during infection by antibody-opsonized virus. PLoS Pathog 11: e1005253 10.1371/journal.ppat.1005253 26506431 PMC4624778

[51] Dai P, Wang W, Cao H, Avogadri F, Dai L, Drexler I, Joyce J, Li X, Chen Z, Merghoub T, (2014) Modified vaccinia virus Ankara triggers type I IFN production in murine conventional dendritic cells via a cGAS/STING-mediated cytosolic DNA-sensing pathway. PLoS Pathog 10: e1003989 10.1371/journal.ppat.1003989 24743339 PMC3990710

[52] Garcia-Belmonte R, Perez-Nunez D, Pittau M, Richt J, Revilla Y (2019) African swine fever virus Armenia/07 virulent strain controls interferon beta production through the cGAS-STING pathway. J Virol 93: e02298-18. 10.1128/jvi.02298-18 30918080 PMC6613762

[53] Zierhut C, Yamaguchi N, Paredes M, Luo J, Carroll T, Funabiki H (2019) The cytoplasmic DNA sensor cGAS promotes mitotic cell death. Cell 178: 302–315.e23. 10.1016/j.cell.2019.05.035 31299200 PMC6693521

[54] Volkman H, Cambier S, Gray E, Stetson D (2019) Tight nuclear tethering of cGAS is essential for preventing autoreactivity. Elife 8: e47491 10.7554/elife.47491 31808743 PMC6927687

[55] Sutterlin C, Hsu P, Mallabiabarrena A, Malhotra V (2002) Fragmentation and dispersal of the pericentriolar Golgi complex is required for entry into mitosis in mammalian cells. Cell 109: 359–369. 10.1016/s0092-8674(02)00720-1 12015985

[56] Tang D, Wang Y (2013) Cell cycle regulation of Golgi membrane dynamics. Trends Cell Biol 23: 296–304. 10.1016/j.tcb.2013.01.008 23453991 PMC3665639

[57] Boukamp P, Petrussevska R, Breitkreutz D, Hornung J, Markham A, Fusenig N (1988) Normal keratinization in a spontaneously immortalized aneuploid human keratinocyte cell line. J Cell Biol 106: 761–771. 10.1083/jcb.106.3.761 2450098 PMC2115116

[58] Bernard E, Hamel R, Neyret A, Ekchariyawat P, Moles J, Simmons G, Chazal N, Despres P, Misse D, Briant L (2015) Human keratinocytes restrict chikungunya virus replication at a post-fusion step. Virology 476: 1–10. 10.1016/j.virol.2014.11.013 25496825

[59] Griffin L, Cicchini L, Xu T, Pyeon D (2014) Human keratinocyte cultures in the investigation of early steps of human papillomavirus infection. Methods Mol Biol 1195: 219–238. 10.1007/7651_2013_49 24281871 PMC4076347

[60] Kim J, Seong R, Son S, Shin O (2019) Insights into ZIKV-mediated innate immune responses in human dermal fibroblasts and epidermal keratinocytes. J Invest Dermatol 139: 391–399. 10.1016/j.jid.2018.07.038 30218650

[61] Lim P, Behr M, Chadwick C, Shi P, Bernard K (2011) Keratinocytes are cell targets of West Nile virus in vivo. J Virol 85: 5197–5201. 10.1128/jvi.02692-10 21367890 PMC3126165

[62] Lopez-Gonzalez M, Meza-Sanchez D, Garcia-Cordero J, Bustos-Arriaga J, Velez-Del Valle C, Marsch-Moreno M, Castro-Jimenez T, Flores-Romo L, Santos-Argumedo L, Gutierrez-Castaneda B, (2018) Human keratinocyte cultures (HaCaT) can be infected by DENV, triggering innate immune responses that include IFNlambda and LL37. Immunobiology 223: 608–617. 10.1016/j.imbio.2018.07.006 30007822

[63] Puiprom O, Morales Vargas R, Potiwat R, Chaichana P, Ikuta K, Ramasoota P, Okabayashi T (2013) Characterization of chikungunya virus infection of a human keratinocyte cell line: Role of mosquito salivary gland protein in suppressing the host immune response. Infect Genet Evol 17: 210–215. 10.1016/j.meegid.2013.04.005 23583544

[64] Rahn E, Petermann P, Hsu M, Rixon F, Knebel-Morsdorf D (2011) Entry pathways of herpes simplex virus type 1 into human keratinocytes are dynamin- and cholesterol-dependent. PLoS One 6: e25464 10.1371/journal.pone.0025464 22022400 PMC3192061

[65] Kisich K, Howell M, Boguniewicz M, Heizer H, Watson N, Leung D (2007) The constitutive capacity of human keratinocytes to kill Staphylococcus aureus is dependent on beta-defensin 3. J Invest Dermatol 127: 2368–2380. 10.1038/sj.jid.5700861 17460726

[66] Petrucelli M, Peronni K, Sanches P, Komoto T, Matsuda J, Silva Junior W, Beleboni R, Martinez-Rossi N, Marins M, Fachin A (2018) Dual RNA-seq analysis of trichophyton rubrum and HaCat keratinocyte Co-culture highlights important genes for fungal-host interaction. Genes (Basel) 9: 362 10.3390/genes9070362 PMC6070946 30029541

[67] Almine J, O’Hare C, Dunphy G, Haga I, Naik R, Atrih A, Connolly D, Taylor J, Kelsall I, Bowie A, (2017) IFI16 and cGAS cooperate in the activation of STING during DNA sensing in human keratinocytes. Nat Commun 8: 14392 10.1038/ncomms14392 28194029 PMC5316833

[68] Dunphy G, Flannery S, Almine J, Connolly D, Paulus C, Jonsson K, Jakobsen M, Nevels M, Bowie A, Unterholzner L (2018) Non-canonical activation of the DNA sensing adaptor STING by ATM and IFI16 mediates NF-kappaB signaling after nuclear DNA damage. Mol Cell 71: 745–760.e5. 10.1016/j.molcel.2018.07.034 30193098 PMC6127031

[69] Kim J, Park S, Seo S, Lee C, Shin O (2017) STING is involved in antiviral immune response against VZV infection via the induction of type I and III IFN pathways. J Invest Dermatol 137: 2101–2109. 10.1016/j.jid.2017.03.041 28647346

[70] Olagnier D, Brandtoft A, Gunderstofte C, Villadsen N, Krapp C, Thielke A, Laustsen A, Peri S, Hansen A, Bonefeld L, (2018) Nrf2 negatively regulates STING indicating a link between antiviral sensing and metabolic reprogramming. Nat Commun 9: 3506 10.1038/s41467-018-05861-7 30158636 PMC6115435

[71] Skouboe M, Knudsen A, Reinert L, Boularan C, Lioux T, Perouzel E, Thomsen M, Paludan S (2018) STING agonists enable antiviral cross-talk between human cells and confer protection against genital herpes in mice. PLoS Pathog 14: e1006976 10.1371/journal.ppat.1006976 29608601 PMC5897032

[72] Cole N, Sciaky N, Marotta A, Song J, Lippincott-Schwartz J (1996) Golgi dispersal during microtubule disruption: Regeneration of Golgi stacks at peripheral endoplasmic reticulum exit sites. Mol Biol Cell 7: 631–650. 10.1091/mbc.7.4.631 8730104 PMC275914

[73] Helms J, Rothman J (1992) Inhibition by brefeldin A of a Golgi membrane enzyme that catalyses exchange of guanine nucleotide bound to ARF. Nature 360: 352–354. 10.1038/360352a0 1448152

[74] Saenz J, Sun W, Chang J, Li J, Bursulaya B, Gray N, Haslam D (2009) Golgicide A reveals essential roles for GBF1 in Golgi assembly and function. Nat Chem Biol 5: 157–165. 10.1038/nchembio.144 19182783 PMC3500152

[75] Gui X, Yang H, Li T, Tan X, Shi P, Li M, Du F, Chen Z (2019) Autophagy induction via STING trafficking is a primordial function of the cGAS pathway. Nature 567: 262–266. 10.1038/s41586-019-1006-9 30842662 PMC9417302

[76] Kawai T, Takahashi K, Sato S, Coban C, Kumar H, Kato H, Ishii K, Takeuchi O, Akira S (2005) IPS-1, an adaptor triggering RIG-I- and Mda5-mediated type I interferon induction. Nat Immunol 6: 981–988. 10.1038/ni1243 16127453

[77] Yoneyama M, Kikuchi M, Natsukawa T, Shinobu N, Imaizumi T, Miyagishi M, Taira K, Akira S, Fujita T (2004) The RNA helicase RIG-I has an essential function in double-stranded RNA-induced innate antiviral responses. Nat Immunol 5: 730–737. 10.1038/ni1087 15208624

[78] Farmaki T, Ponnambalam S, Prescott A, Clausen H, Tang B, Hong W, Lucocq J (1999) Forward and retrograde trafficking in mitotic animal cells. ER-Golgi transport arrest restricts protein export from the ER into COPII-coated structures. J Cell Sci 112: 589–600. 9973594 10.1242/jcs.112.5.589

[79] Kreiner T, Moore H (1990) Membrane traffic between secretory compartments is differentially affected during mitosis. Cell Regul 1: 415–424. 10.1091/mbc.1.5.415 2099191 PMC361519

[80] Prescott A, Farmaki T, Thomson C, James J, Paccaud J, Tang B, Hong W, Quinn M, Ponnambalam S, Lucocq J (2001) Evidence for prebudding arrest of ER export in animal cell mitosis and its role in generating Golgi partitioning intermediates. Traffic 2: 321–335. 10.1034/j.1600-0854.2001.002005321.x 11350628

[81] Warren G, Featherstone C, Griffiths G, Burke B (1983) Newly synthesized G protein of vesicular stomatitis virus is not transported to the cell surface during mitosis. J Cell Biol 97: 1623–1628. 10.1083/jcb.97.5.1623 6355124 PMC2112686

[82] Hughes H, Stephens D (2010) Sec16A defines the site for vesicle budding from the endoplasmic reticulum on exit from mitosis. J Cell Sci 123: 4032–4038. 10.1242/jcs.076000 21045114 PMC2987439

[83] Ward T, Polishchuk R, Caplan S, Hirschberg K, Lippincott-Schwartz J (2001) Maintenance of Golgi structure and function depends on the integrity of ER export. J Cell Biol 155: 557–570. 10.1083/jcb.200107045 11706049 PMC2198855

[84] Luteijn R, Zaver S, Gowen B, Wyman S, Garelis N, Onia L, McWhirter S, Katibah G, Corn J, Woodward J, (2019) SLC19A1 transports immunoreactive cyclic dinucleotides. Nature 573: 434–438. 10.1038/s41586-019-1553-0 31511694 PMC6785039

[85] Ritchie C, Cordova A, Hess G, Bassik M, Li L (2019) SLC19A1 is an importer of the immunotransmitter cGAMP. Mol Cell 75: 372–381.e5. 10.1016/j.molcel.2019.05.006 31126740 PMC6711396

[86] Zhou C, Chen X, Planells-Cases R, Chu J, Wang L, Cao L, Li Z, Lopez-Cayuqueo K, Xie Y, Ye S, (2020) Transfer of cGAMP into bystander cells via LRRC8 volume-regulated anion channels augments STING-mediated interferon responses and anti-viral immunity. Immunity 52: 767–781.e6. 10.1016/j.immuni.2020.03.016 32277911

[87] Ablasser A, Schmid-Burgk J, Hemmerling I, Horvath G, Schmidt T, Latz E, Hornung V (2013) Cell intrinsic immunity spreads to bystander cells via the intercellular transfer of cGAMP. Nature 503: 530–534. 10.1038/nature12640 24077100 PMC4142317

[88] Fykerud T, Knudsen L, Totland M, Sorensen V, Dahal-Koirala S, Lothe R, Brech A, Leithe E (2016) Mitotic cells form actin-based bridges with adjacent cells to provide intercellular communication during rounding. Cell Cycle 15: 2943–2957. 10.1080/15384101.2016.1231280 27625181 PMC5105929

[89] Odle R, Walker S, Oxley D, Kidger A, Balmanno K, Gilley R, Okkenhaug H, Florey O, Ktistakis N, Cook S (2019) An mTORC1-to-CDK1 switch maintains autophagy suppression during mitosis. Mol Cell 77: 228–240. 10.1016/j.molcel.2019.10.016. 31733992 PMC6964153

[90] Willson J (2019) Mitosis flips the switch on autophagy control. Nat Rev Mol Cell Biol 21: 4–5. 10.1038/s41580-019-0196-1 31758162

[91] Eskelinen E, Prescott A, Cooper J, Brachmann S, Wang L, Tang X, Backer J, Lucocq J (2002) Inhibition of autophagy in mitotic animal cells. Traffic 3: 878–893. 10.1034/j.1600-0854.2002.31204.x 12453151

[92] Mathiassen S, De Zio D, Cecconi F (2017) Autophagy and the cell cycle: A complex landscape. Front Oncol 7: 51 10.3389/fonc.2017.00051 28409123 PMC5374984

[93] Zhong L, Hu M, Bian L, Liu Y, Chen Q, Shu H (2020) Phosphorylation of cGAS by CDK1 impairs self-DNA sensing in mitosis. Cell Discov 6: 26 10.1038/s41421-020-0162-2 32351706 PMC7186227

[94] Cervigni R, Bonavita R, Barretta M, Spano D, Ayala I, Nakamura N, Corda D, Colanzi A (2015) JNK2 controls fragmentation of the Golgi complex and the G2/M transition through phosphorylation of GRASP65. J Cell Sci 128: 2249–2260. 10.1242/jcs.164871 25948586

[95] Colanzi A, Hidalgo Carcedo C, Persico A, Cericola C, Turacchio G, Bonazzi M, Luini A, Corda D (2007) The Golgi mitotic checkpoint is controlled by BARS-dependent fission of the Golgi ribbon into separate stacks in G2. EMBO J 26: 2465–2476. 10.1038/sj.emboj.7601686 17431394 PMC1868899

[96] Corda D, Barretta M, Cervigni R, Colanzi A (2012) Golgi complex fragmentation in G2/M transition: An organelle-based cell-cycle checkpoint. IUBMB Life 64: 661–670. 10.1002/iub.1054 22730233

[97] Canton J, Kima P (2012) Interactions of pathogen-containing compartments with the secretory pathway. Cell Microbiol 14: 1676–1686. 10.1111/cmi.12000 22862745

[98] Machamer C (2015) The Golgi complex in stress and death. Front Neurosci 9: 421 10.3389/fnins.2015.00421 26594142 PMC4635215

[99] Mousnier A, Swieboda D, Pinto A, Guedan A, Rogers A, Walton R, Johnston S, Solari R (2014) Human rhinovirus 16 causes Golgi apparatus fragmentation without blocking protein secretion. J Virol 88: 11671–11685. 10.1128/jvi.01170-14 25100828 PMC4178721

[100] Heuer D, Rejman Lipinski A, Machuy N, Karlas A, Wehrens A, Siedler F, Brinkmann V, Meyer T (2009) Chlamydia causes fragmentation of the Golgi compartment to ensure reproduction. Nature 457: 731–735. 10.1038/nature07578 19060882

[101] Pierini R, Cottam E, Roberts R, Wileman T (2009) Modulation of membrane traffic between endoplasmic reticulum, ERGIC and Golgi to generate compartments for the replication of bacteria and viruses. Semin Cell Dev Biol 20: 828–833. 10.1016/j.semcdb.2009.03.015 19508853 PMC7110581

[102] Manders E, Verbeek F, Aten J (1993) Measurement of co-localization of objects in dual-colour confocal images. J Microsc 169: 375–382. 10.1111/j.1365-2818.1993.tb03313.x 33930978

[103] Bolte S, Cordelieres F (2006) A guided tour into subcellular colocalization analysis in light microscopy. J Microsc 224: 213–232. 10.1111/j.1365-2818.2006.01706.x 17210054

[104] Schneider C, Rasband W, Eliceiri K (2012) NIH image to ImageJ: 25 years of image analysis. Nat Methods 9: 671–675. 10.1038/nmeth.2089 22930834 PMC5554542

[105] McDonald J, Dunn K (2013) Statistical tests for measures of colocalization in biological microscopy. J Microsc 252: 295–302. 10.1111/jmi.12093 24117417 PMC4428547

