# Supplementary figures and images for: Attenuation of cGAS/STING activity during mitosis
Source: Life Sci Alliance. 2020 Jul 13;3(9):e201900636. doi: 10.26508/lsa.201900636 (PMC7368095; doi:10.26508/lsa.201900636)

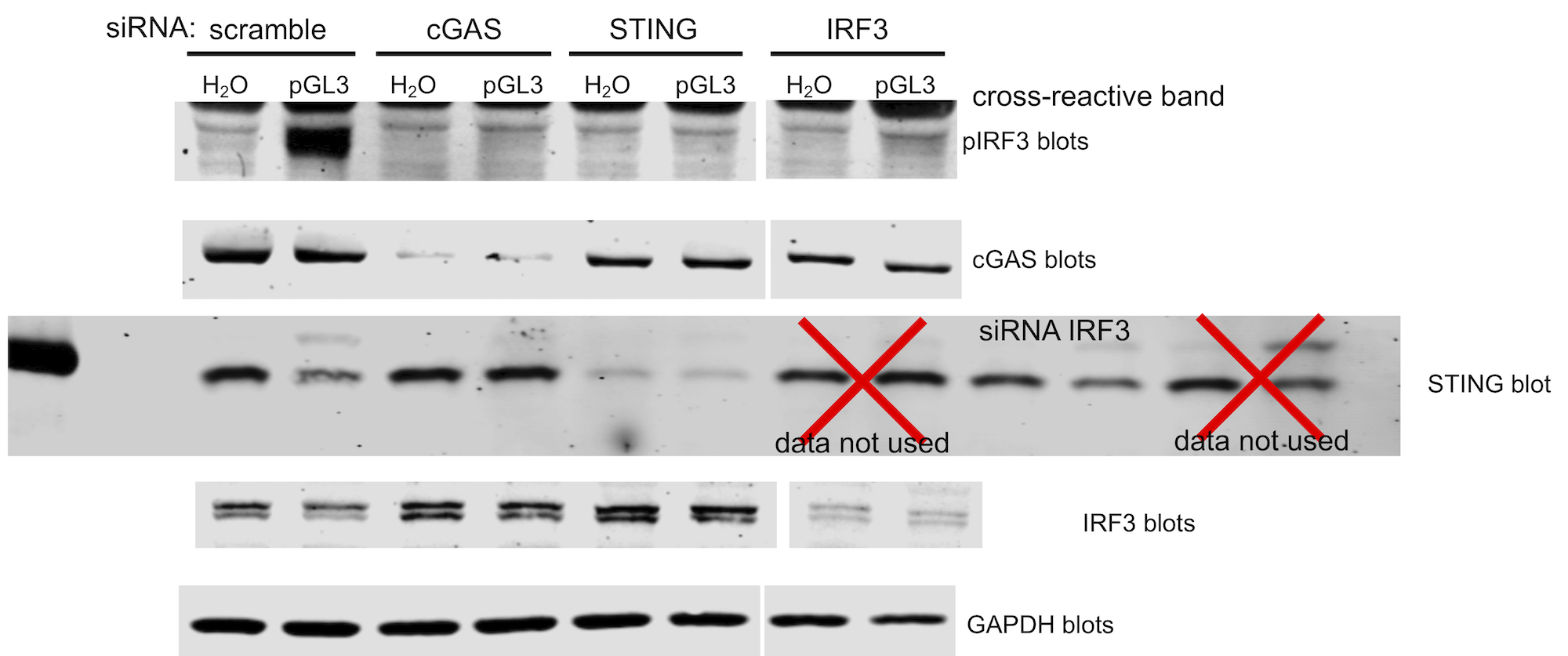

Supplement: Supplementary file 1 [file LSA-2019-00636_SdataF1.tif]

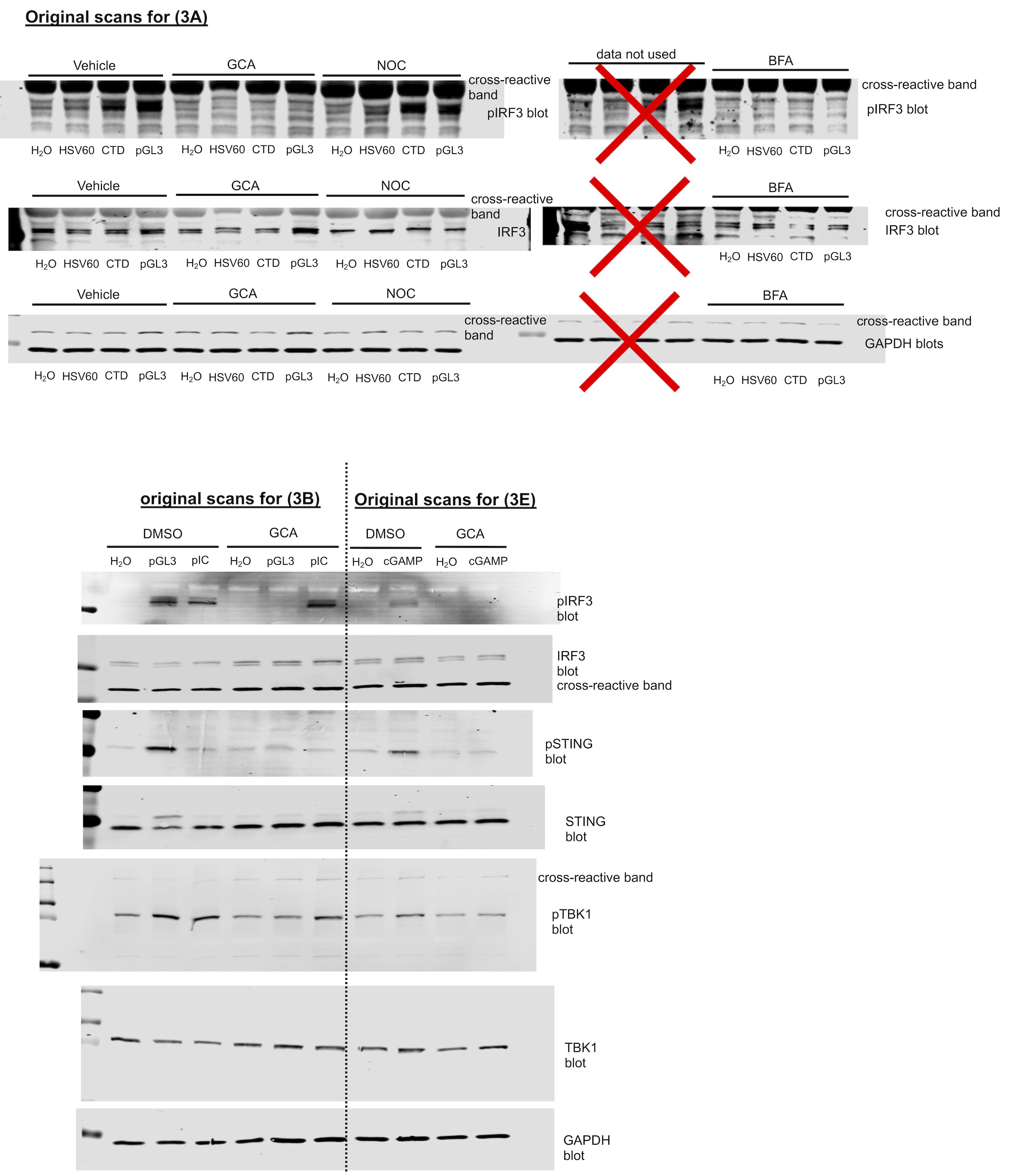

Supplement: Supplementary file 2 [file LSA-2019-00636_SdataF3.tif]

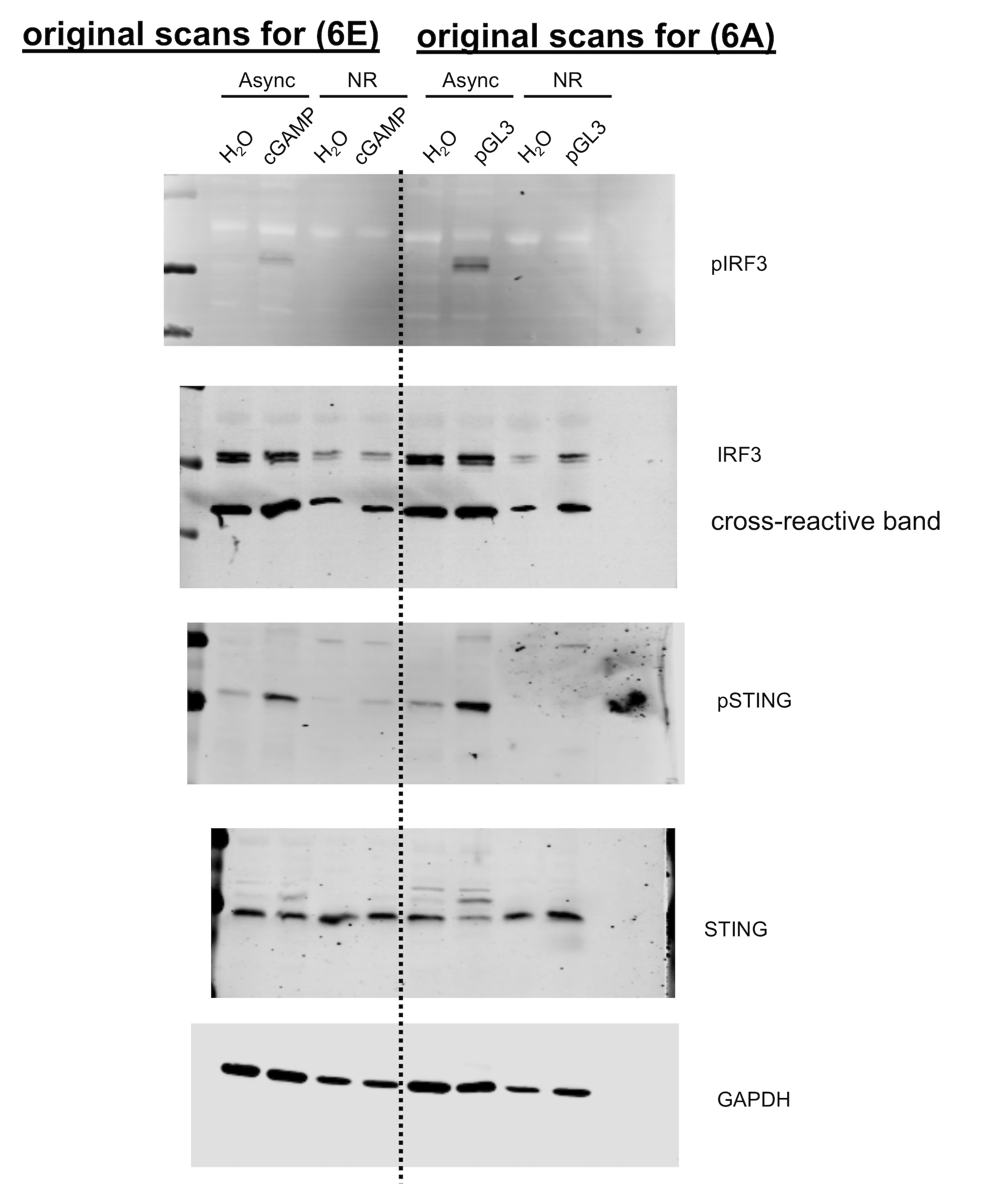

Supplement: Supplementary file 3 [file LSA-2019-00636_SdataF6.tif]
